# The GRE over the entire range of scores lacks predictive ability for PhD outcomes in the biomedical sciences

**DOI:** 10.1371/journal.pone.0201634

**Published:** 2019-03-21

**Authors:** Linda Sealy, Christina Saunders, Jeffrey Blume, Roger Chalkley

**Affiliations:** 1 Office of the Dean of Basic Sciences, School of Medicine, Vanderbilt University, Nashville, Tennessee, United States of America; 2 Department of Molecular Physiology and Biophysics, School of Medicine, Vanderbilt University, Nashville, Tennessee, United States of America; 3 Department of Biostatistics, School of Medicine, Vanderbilt University, Nashville, Tennessee, United States of America; Universidade de Mogi das Cruzes, BRAZIL

## Abstract

The association between GRE scores and academic success in graduate programs is currently of national interest. GRE scores are often assumed to be predictive of student success in graduate school. However, we found no such association in admission data from Vanderbilt’s Initiative for Maximizing Student Diversity (IMSD), which recruited historically underrepresented students for graduate study in the biomedical sciences at Vanderbilt University spanning a wide range of GRE scores. This study avoids the typical biases of most GRE investigations of performance where primarily high-achievers on the GRE were admitted. GRE scores, while collected at admission, were not used or consulted for admission decisions and comprise the full range of percentiles, from 1% to 91%. We report on the 32 students recruited to the Vanderbilt IMSD from 2007–2011, of which 28 completed the PhD to date. While the data set is not large, the predictive trends between GRE and long-term graduate outcomes (publications, first author publications, time to degree, predoctoral fellowship awards, and faculty evaluations) are remarkably null and there is sufficient precision to rule out even mild relationships between GRE and these outcomes. Career outcomes are encouraging; many students are in postdocs, and the rest are in regular stage-appropriate career environments for such a cohort, including tenure track faculty, biotech and entrepreneurship careers.

## Introduction

Recently Moneta-Kohler et al. [1] published a detailed statistical analysis of the lack of ability of the GRE to predict performance in graduate school in the biomedical research arena at Vanderbilt. A similar study was published by Hall et al. [2] from the University of North Carolina Chapel Hill. However, there was a limitation to the overall conclusions in that the range of GRE scores did not cover scores lower than approximately 50%. In order to test if such a limitation impacted the predictive ability of the GRE, we would need to admit students for whom we had GRE information, but where the admitted students covered the entire range of scores with no bias or cut-off (deliberate or otherwise) in the level of the score. This is a difficult requirement, as admissions committees normally do not pursue applicants with very low GRE scores, even if other aspects of the application might appear to be competitive. We are aware that a fairly significant number of schools are electing to not use GRE scores at all in making admissions decisions [3]. Other schools may be considering whether or not to require GRE scores, but have not yet taken action. All of these schools would most likely benefit if there were to be an experiment in which we assayed the predictive ability of the GRE scores over the entire range of scores.

We report that we have performed this natural experiment with GRE scores covering the range from 1st to 91st percentile, in an approach where the scores, although submitted as part of the application, were not considered in the selection of incoming graduate students. This came about in the following way. In 2007 Vanderbilt was awarded an NIGMS-funded IMSD program with the goal of increasing the number of students from underrepresented groups completing PhDs in the biomedical sciences. This program was a redesign of our previous IMSD post baccalaureate program in response to the NIH stipulation in 2006 that students in the program had to be matriculated as graduate students, not post baccalaureates. We were aware that increasing the number of historically underrepresented (UR) students in our PhD programs might result in another school(s) not enrolling these students, and the overall pool of UR PhD trainees would remain static. This was because at that time the pool of high qualified UR students, when quantitative metrics (GRE, GPA) were a key driver of the assessment, was in insufficient supply. The authors had already collected data (unpublished) over a ten-year period indicating that for underrepresented students at least the GRE at the levels usually expected for admission offered no guidance in terms of achievements of long term PhD training goals. Consequently, we decided that removing the barrier of GRE scores to admission would actually lead to an overall increase in historically underrepresented PhD trainees.

Therefore, in 2007 the Vanderbilt IMSD program adopted a fully holistic approach to admissions. The GRE scores were recorded as a required part of the application process, but they were essentially ignored by the IMSD admissions committee, which operated in a separate fashion from our regular interdisciplinary graduate program (IGP) admissions committee. This resulted in a group of students who were eligible for IMSD support (as defined by NIGMS) admitted with GRE scores over the full range (1–90% GRE-V and 11–91% GRE-Q). Details of the GRE-tolerant, fully holistic approach are presented below, but relied heavily on letters of recommendation, personal statements and interviews. If these factors were strong, no GRE score was too low to be admitted.

Over the next four years, 32 students were admitted in this fashion from 2007–2011. We are able to evaluate the outcomes of admissions strategies which cover the entire GRE range (including both very high and low scores) under conditions in which the admissions process operated obliviously to the scores themselves. The measures we have used to evaluate outcomes performance in biomedical research were also used in our previous report [1] on the lack of predictive value of the GRE. These include: number of first or other order author papers, receipt of competitive fellowship awards, time to degree, a detailed faculty evaluation at the time of graduation, and an initial review of career development in scientific areas. Of the 32 URM students who participated in the IMSD program over this time period, 30 have now graduated (28 PhD, 2 MS) with two students dropping out early as a consequence of health problems. The attrition of these two students was beyond our control; however, of the remaining 30 students, 93% (28/30 students) completed the Ph.D. We present here the outcomes of the 28 PhD graduates and the relationship of these outcomes to their GRE scores.

## Materials and methods

**Fig 1:**
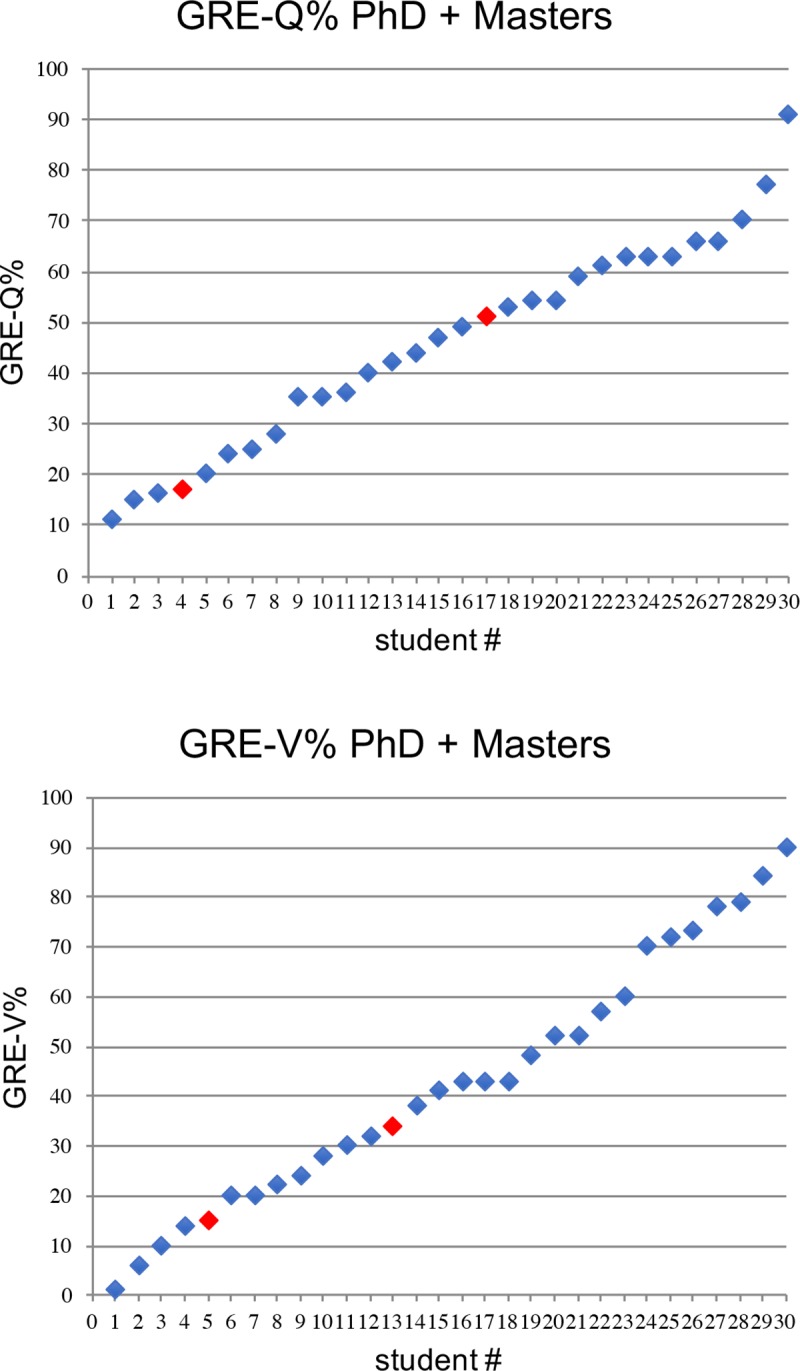
GRE Quantitative and verbal scores of IMSD students matriculating from 2007–11 who completed a PhD or a Master’s degree. Top panel depicts GRE-Q% and lower panel depicts GRE-V% for 30 students who completed either a PhD (blue symbols) or Master’s degree (red symbols).

**Fig 2:**
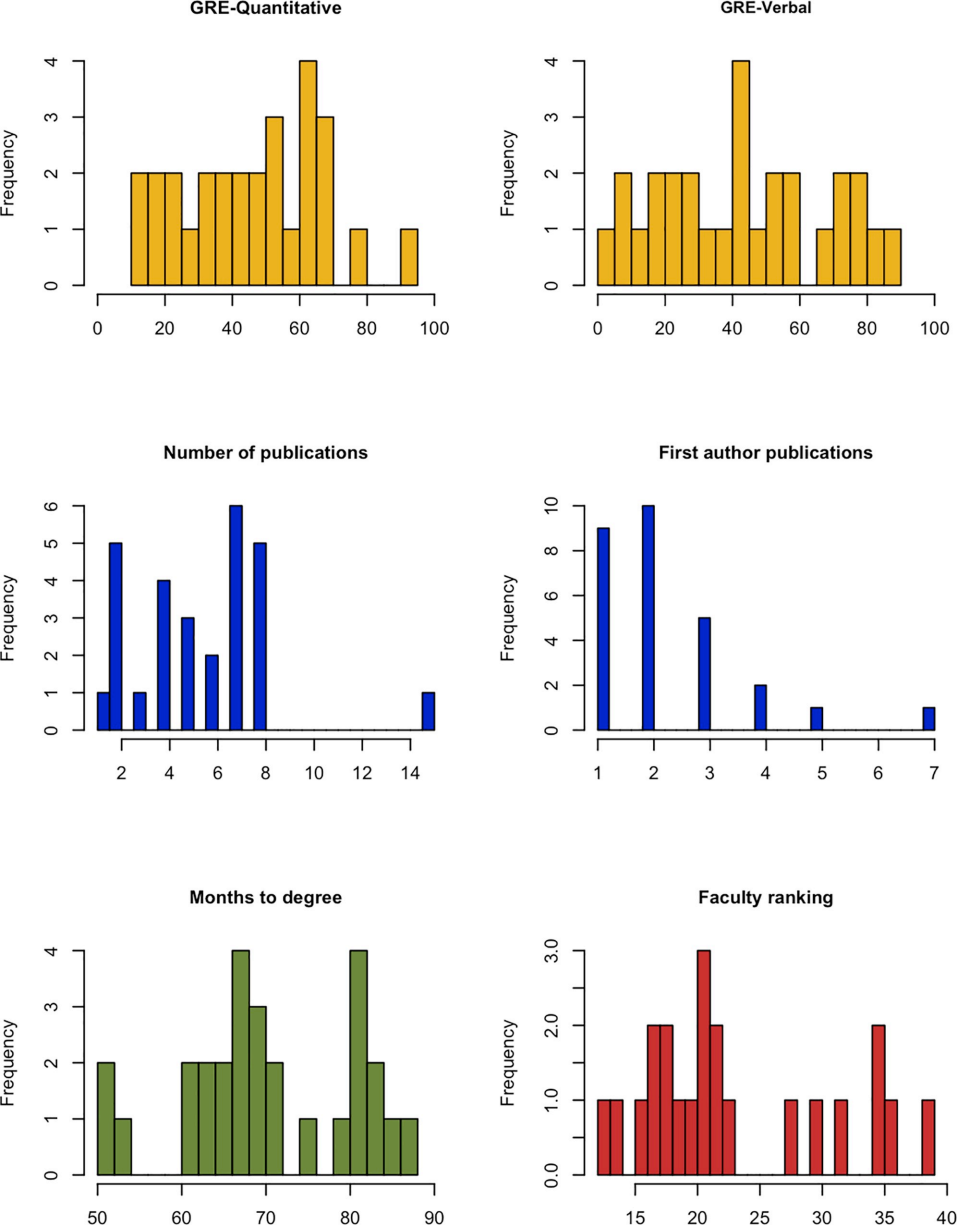
Histograms of outcomes data. Frequencies of GRE scores (Q% and V%), number of publications, months to degree, and faculty ranking are shown as indicated.

GRE (Quantitative and Verbal) scores and academic performance data from 28 IMSD students who matriculated from 2007 to 2011 were collected and examined. Academic performance outcomes of interest were: time elapsed in program (i.e., months to degree), number of publications, number of first-author publications, fellowship status (any or F31), Vanderbilt faculty ranking (10 = best, 50 = worst). Table 1 provides the list of attributes and competencies used in the faculty ranking, and for the remaining criteria, Table 2 provides univariate summaries (e.g., mean, median, standard deviation, inter-quartile range) of these variables. Fig 1 presents the GRE scores for 30 URM students admitted into the graduate program in the biomedical sciences at Vanderbilt from 2007–2011 who completed either a PhD or a MS degree. Fig 2 presents histograms of the continuous outcome variables. Regression modeling was used to assess the degree of association between GRE outcomes and academic outcomes. Specifically, Poisson regression was used to model publication counts (accounting for length of time in the program), months to degree, and faculty ranking. Logistic regression was used to model receipt of fellowship. For all models, we report point estimates, model robust standard errors, and 95% confidence intervals (CIs). We plot each performance measure as a function of GRE scores and include the fitted regression line as well as a locally weighted scatterplot smoother (lowess) line to visually assess linearity assumptions and model fit. Confidence intervals were plotted to demonstrate the degree of precision afforded by the data at the 95% level. Any relationship between GRE scores and outcomes would be captured in the slope of these regression lines. *While it is not possible to prove the null hypothesis that GRE scores and outcomes are not related*, *it is possible to provide an upper bound on the largest potential association*. The 95% CIs provide this boundary and comprise the set of associations supported by the data. As we will see from the data, despite the small sample size, these CIs do not support mild or strong associations between GRE scores and outcomes. For a sensitivity analysis, we compared academic outcomes between the first quartile and the fourth quartile of GRE scores. If any association were present, such an analysis should at least yield exaggerated point estimates of the association effect.

**Table 1:** Faculty rating of student at exit.

1. Ability to handle classwork needed for your PhD program
2. Drive and determination
3. Creativity and imagination in terms of experimental design and interpretation
4. Technical ability
5. Keeping up with the literature
6. Output–translating observations into a presentable paper
7. Ability to write creatively
8. Leadership in the lab and department
9. Trajectory
10. Overall assessment as a productive scientist

Faculty mentors were asked to score student upon PhD completion using a scale of 1–5 as follows

1-outstanding; 2-excellent; 3-good; 4-fair; 5-poor. The faculty rating is the sum of the scores for 10 questions.

**Table 2:** Summary statistics of GRE data.

Summary statistics of GRE data		
**Variable**	**N**	**Summary**
GRE Quantitative	28	48.0 [33.2, 63.0]
GRE Verbal	28	43.0 [23.5, 62.5]
No. of publications	28	5.50 [3.75, 7.00]
No. of first author pubs	28	2 [1, 3]
Any fellowship	28	43% (12)
F31 fellowship	28	29% (8)
Other fellowship: 0	28	86% (24)
1		11% (3)
2		4% (1)
Faculty ranking	22	21.0 [18.0, 29.5]
Months to degree	28	68.6 [64.8, 81.4]

Summary Statistics from the 28 students in the analysis set. For continuous variables, *a [b c]* represent the median *a*, the lower quartile *b*, and the upper quartile *c*. *N* is the number of non–missing values. Numbers after percents are frequencies.

## Results

In Fig 1 we report the range of GRE scores among the 30 URM students admitted into the graduate program in the biomedical sciences at Vanderbilt from 2007 through 2011 and who completed a PhD or a MS degree. The admission decisions for these students during this time period was determined by the IMSD admissions committee, and although the student’s GRE score was recorded in our databases, it has only been used for outcomes studies long after the admissions event. The GRE-tolerant nature of our approach is validated by the range of GRE scores among this group of students. Scores varied across the spectrum for students who were admitted in response to a detailed analysis of the committee’s assessment of the likelihood of the student’s success in research. The committee’s assessment was based primarily on the non-quantitative components of the application, including a close reading of the letters of recommendation and the student’s personal statement. The student’s transcript was evaluated, primarily to assess adequate coursework preparation for biomedical PhD coursework. A wide range of GPAs were accepted. We sought to place the overall and science GPAs in the context of the college or university and the life events of the applicant. For example, students with extensive work and/or family responsibilities might reasonably be expected to end up with lower GPAs due to time demands. The lowest GPA accepted among this group of students was 1.8. Finally, all students were invited to campus for an interview visit that was also given significant consideration.

From the 30 students with GRE scores shown in Fig 1, 93% (28 students) have graduated with the PhD and 7% (2 students) left with an MS degree. Two students who have recently completed the PhD are currently looking for their next position. However, of the remaining26 students who completed the PhD, 85% (22 students) continued to postdoctoral positions. Four students did not continue on to postdocs, choosing instead to move to industry, consulting, medical school, or an academic faculty position. Overall, the outcomes of this cadre of GRE-blind admitted students are strikingly parallel to those of students admitted through the traditional route (using much higher GRE scores) over the same time period [4]. As indicated in Fig 1, we have a wide range of GRE scores among this group. This unusual group provided us with a means to test the predictive value of GRE scores over a much wider range than most admissions committees will typically tolerate.

Fig 2 shows histograms of the data from the 28 PhD graduates analyzed in this study: range and frequencies of GRE scores, number of publications, number of first author publications, months to degree, and faculty ranking. The faculty ranking is obtained upon the student’s completion of their Ph.D. The ranking is comprised of the sum of scores for each of ten questions, listed in Table 1. The questions cover a range of areas that are often informally assessed as measures of developing into a successful, independent scientist; many would fall into the area of the social/emotional learning skillset. We ask the PhD faculty mentor to score their newly-minted PhD student from one to five, with one being best. Thus, the top ranking possible is a 10, if the student received a score of one for each of the ten questions. Student rankings ranged from 12 to 39 with a median of 21. The other metrics are self-explanatory, with number of publications ranging from one to fifteen (median = 5.5) and first author publications from one to six (median = 2). These metrics are actually very similar to those for the non-IMSD students who were admitted over the same 2007–11 time period by the IGP admissions committee using the traditional process including GRE scores. The 209 students in this traditional cohort had a median number of publications of 5 and median first author publications of 2 (see S6 Table and S1 Fig for details.) Note that students are expected to publish at least one first author paper as a requirement for the PhD in most of our biomedical sciences PhD granting programs. The time to degree for the 28 students admitted in a GRE-tolerant manner ranged from slightly more than 4 years, to just over 7 years (median = 5.7 years). In addition to the data shown in Fig 2, we also included whether or not the student obtained an individual fellowship in a national competition (F31, AHA, DOD, etc) as an additional metric. Summary statistics of the data for this study are presented in Table 2. The hypothesis we test is that GRE scores are associated with future performance in a biomedical graduate program. This association will be measured by the slope in a regression model, to be explained shortly.

### Lack of association between GRE scores and publications

**Fig 3:**
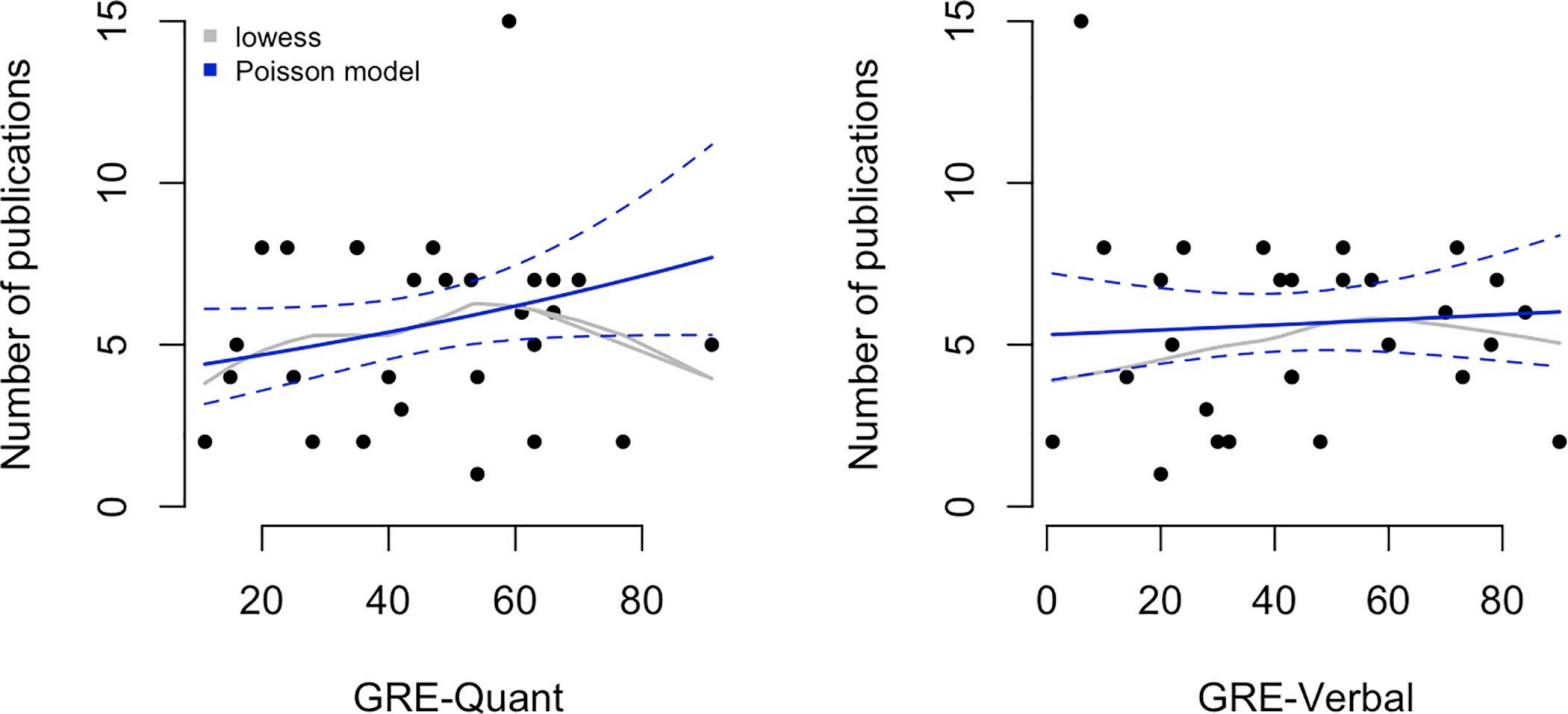
Associations between quantitative and verbal GRE scores and total number of publications. Solid curves show the fitted values from the Poisson regression models (dashed lines are 95% confidence intervals) and the grey curves show lowess smoothers (locally weighted scatterplot smoother).

We modeled the relationship between total number of publications and GRE scores using Poisson regression in Fig 3 for GRE-Q (left panel) and GRE-V (right panel). Solid curves show the fitted values from the regression models (dashed lines are 95% confidence intervals) and the grey curves show lowess smoothers (locally weighted scatterplot smoother). Increasing a student's GRE-Q score by 20 percentage points increases their expected publication rate by 15% (rate ratio = 1.15 with 95% CI 0.952 to 1.39). For instance, students with GRE-Q scores of 40% and 60% are expected to have 5.388 and 6.196 publications, respectively. Interesting, increasing a student's GRE-V score by 20 percentage points increases their expected publication rate by just 2% (rate ratio = 1.028 with 95% CI 0.846 to 1.251) For instance, students with GRE-V scores of 40% and 60% are expected to have 5.61 and 5.768 publications, respectively (a meaningless difference).

**Fig 4:**
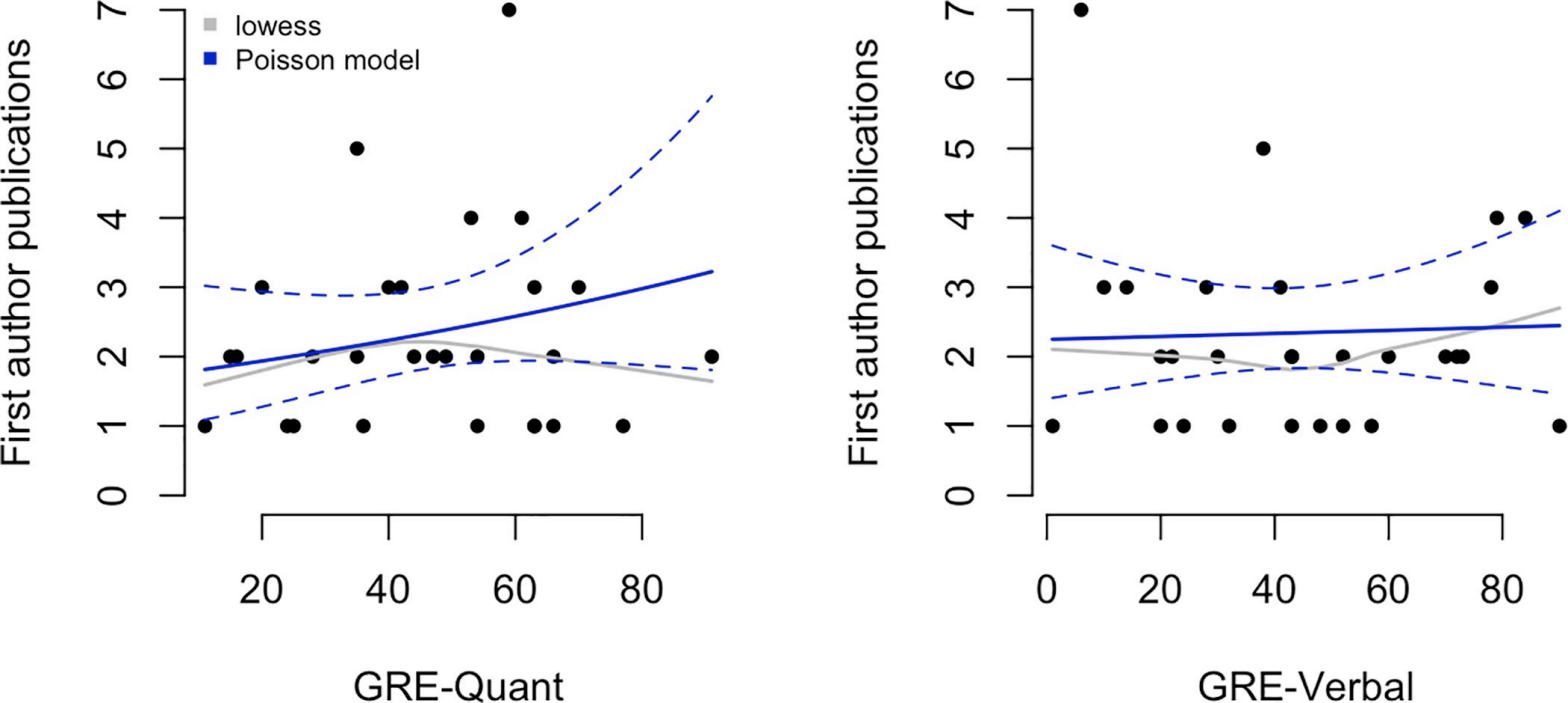
Associations between quantitative and verbal GRE scores and total number of first author publications. Solid curves show the fitted values from the Poisson regression models (dashed lines are 95% confidence intervals) and the grey curves show lowess smoothers (locally weighted scatterplot smoother).

We do not judge either of these minor differences to be scientifically relevant. Similar minor differences were also observed when the total number of first author publications and GRE scores was modeled using Poisson regression in Fig 4. Increasing a student's GRE-V score by 20 percentage points increases their expected publication rate by 0.1% (rate ratio = 1.001 with 95% CI 0.99 to 1.012). For instance, students with GRE-V scores of 40% and 60% are expected to have 2.334 and 2.379 first author publications, respectively. Increasing a student's GRE-Q score by 20 percentage points increases their expected first author publication rate by 15% (rate ratio = 1.154 with 95% CI 0.954 to 1.397). For instance, students with GRE-Q scores of 40% and 60% are expected to have 2.236 and 2.581 first author publications, respectively, which is essentially no difference. We conclude that even when GRE scores below the 20th percentile are in the mix, productivity as measured by the key currency of the scientific enterprise, namely publications–exhibits, at most, very little dependence on GRE score and may well be unrelated in any meaningful sense.

### Lack of association between GRE scores and time to degree

**Fig 5:**
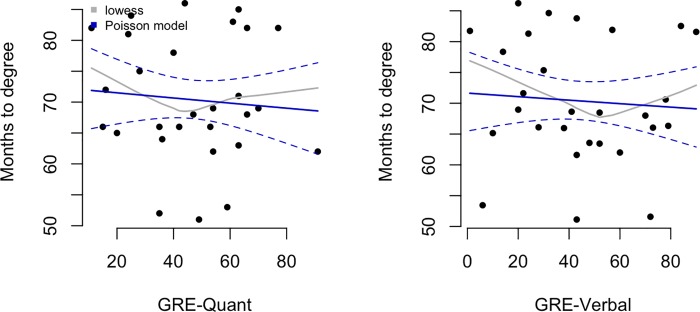
Associations between quantitative and verbal GRE scores and months to degree. Solid curves show the fitted values from the Poisson regression models (dashed lines are 95% confidence intervals) and the grey curves show lowess smoothers (locally weighted scatterplot smoother).

In Fig 5, months to degree is plotted vs either GRE-Q (left panel) or GRE-V (right panel). Again, the solid curve shows the fitted values from the Poisson regression model (dashed lines are 95% confidence intervals) and the grey curve shows a lowess smoother (locally weighted scatterplot smoother). We observe only a very minor correlation between higher GRE scores and shorter time to degree. Increasing either the GRE-Q or GRE-V by 20 percentage points leads to a minor decrease in expected time to degree attainment of 1 month (rate ratio = 0.99 with 95% CI 0.0.997 to 1.002) and (rate ratio = 1 with 95% CI 0.997 to 1.002), respectively. This means that students with GRE-Q scores of 40% and 60% are expected to take 71 months and 70 months to complete their degree, respectively. Likewise, students with GRE-V scores of 40% and 60% are expected to take 71 months and 70 months to complete their degree, respectively.

### Lack of association between GRE scores and fellowships

We are well aware that counting papers, either first author or total, has limitations–especially since neither metric captures the quality and/or impact of the publications. Such parameters are difficult to uniformly measure because they are often very field-specific, and sometimes the impact of research is not fully appreciated for years to come. Therefore, we sought to include individual fellowships obtained as one metric of student quality. We included fellowships that are reviewed nationally by panels of experts, providing a comparison between students in this cohort against students at similar stages of training from other institutions around the country. Predoctoral fellowships obtained by this cohort are included in Table 3.

**Table 3:** Individual fellowships awarded to IMSD students matriculating from 2007–2011.

Fellowship type	number awarded
F31 Ruth L. Kirschstein National Research Service Award (NRSA) Predoctoral Fellowship	8
American Heart Association Predoctoral Fellowship	1
National Science Foundation Graduate Research Fellowship	1
UNCF Merck Graduate Science Research Dissertation Fellowship	1
Department of Defense Prostate Cancer Research Program Predoctoral Fellowship	1

**Fig 6:**
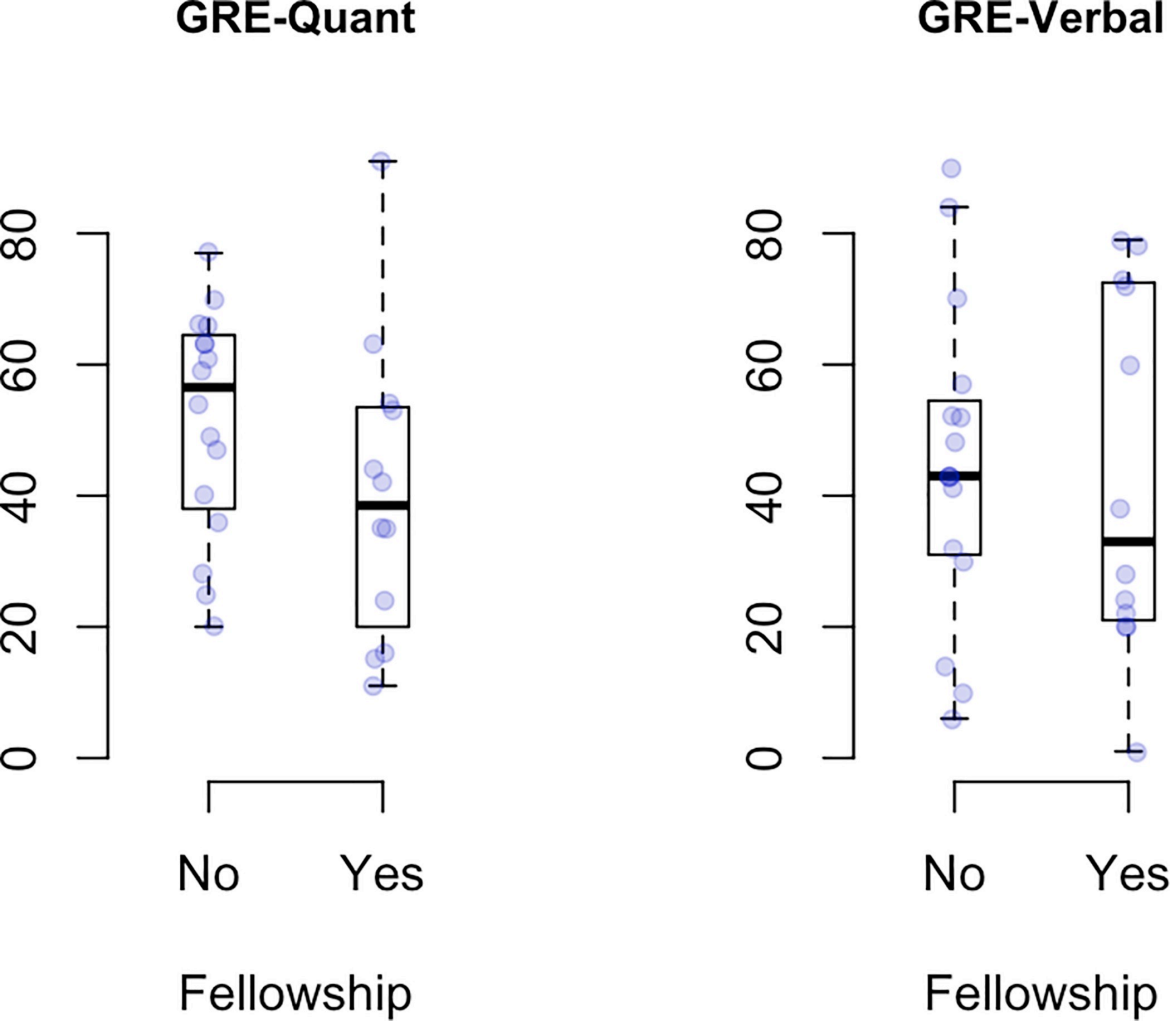
Boxplots of GRE scores stratified by whether or not the students received a fellowship. Data are for fellowships listed in Table 3. The raw data points are overlaid. From bottom to top, the horizontal lines of a boxplot show the minimum GRE score, 25^th^ percentile, median, 75^th^ percentile, and max values in a given group.

**Fig 7:**
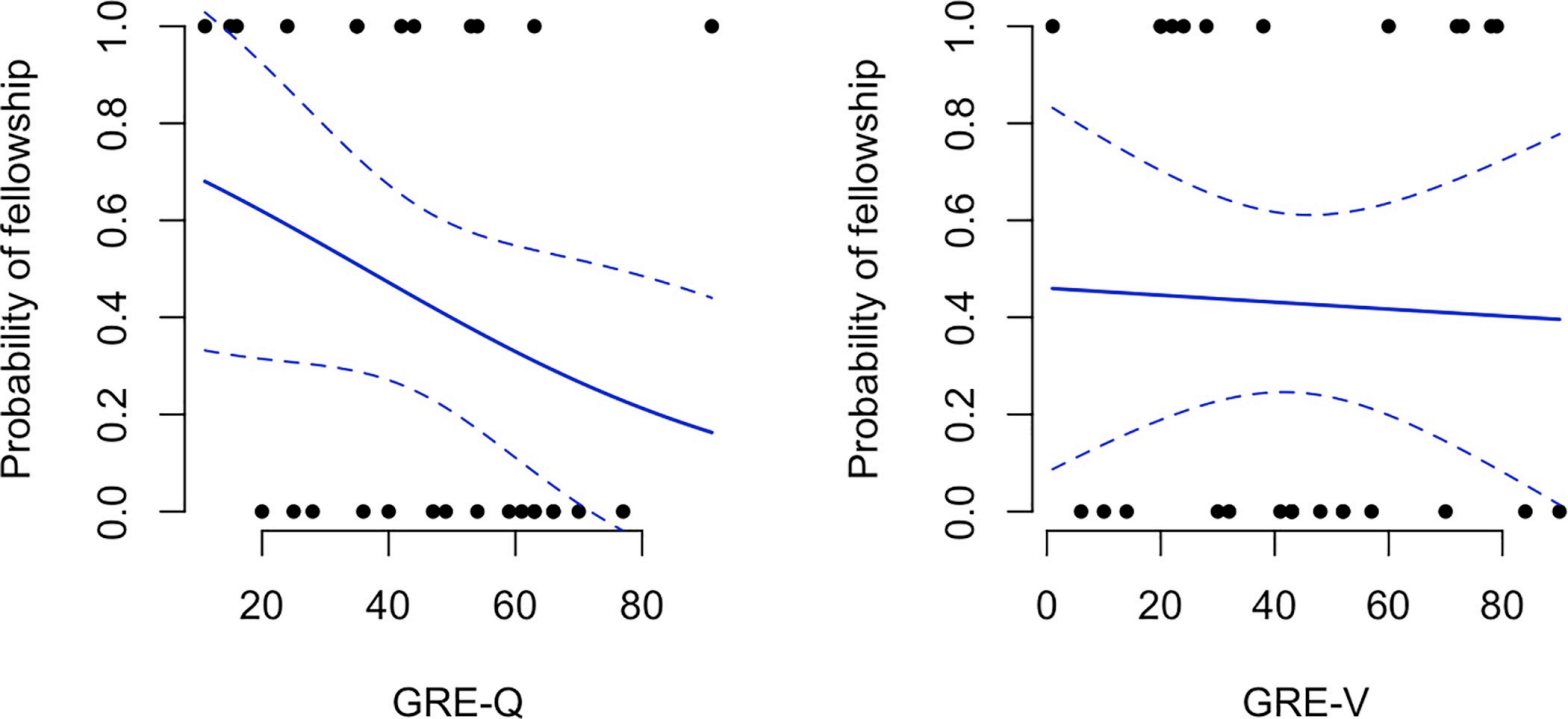
Predicted probability of obtaining a fellowship as a function of GRE scores. Data are for fellowships listed in Table 3. The raw data points are overlaid (0 = No fellowship, 1 = Fellowship).

Boxplots of GRE scores stratified by whether or not students received a fellowship are shown in Fig 6. From bottom to top, the horizontal lines of a boxplot show the min, 25th percentile, median, 75th percentile, and max values in a given group. In Fig 7 the predicted probability of obtaining a fellowship as a function of GRE score is presented. Interestingly, increasing a student’s GRE-Q score by 20 percentage points decreases their odds of receiving a fellowship by 45% (odds ratio = 0.55 with 95% CI 0.217 to 1.392). For instance, the predicted probability of receiving a fellowship for students with GRE-Q scores of 40% and 60% are 47.2% (95% CI 21.7% to 67.3%) and 33% (95% CI is 11.1% to 54.8%), respectively. Alternatively, increasing a student’s GRE-V score by 20 percentage points decreases their odds of receiving a fellowship by just 5.7% (odds ratio = 0.943 with 95% CI 0.507 to 1.754). The predicted probability of receiving a fellowship for students with GRE-V scores of 40% vs. 60% are 43% (24.6%, 61.7%) and 41.7% (19.8%, 63.6%), respectively. We conclude for this data set, that GRE scores have little value in predicting who will receive a fellowship; in fact, for both GRE-Q and GRE-V we observed a negative correlation.

### Lack of association between GRE scores and faculty evaluation

**Fig 8:**
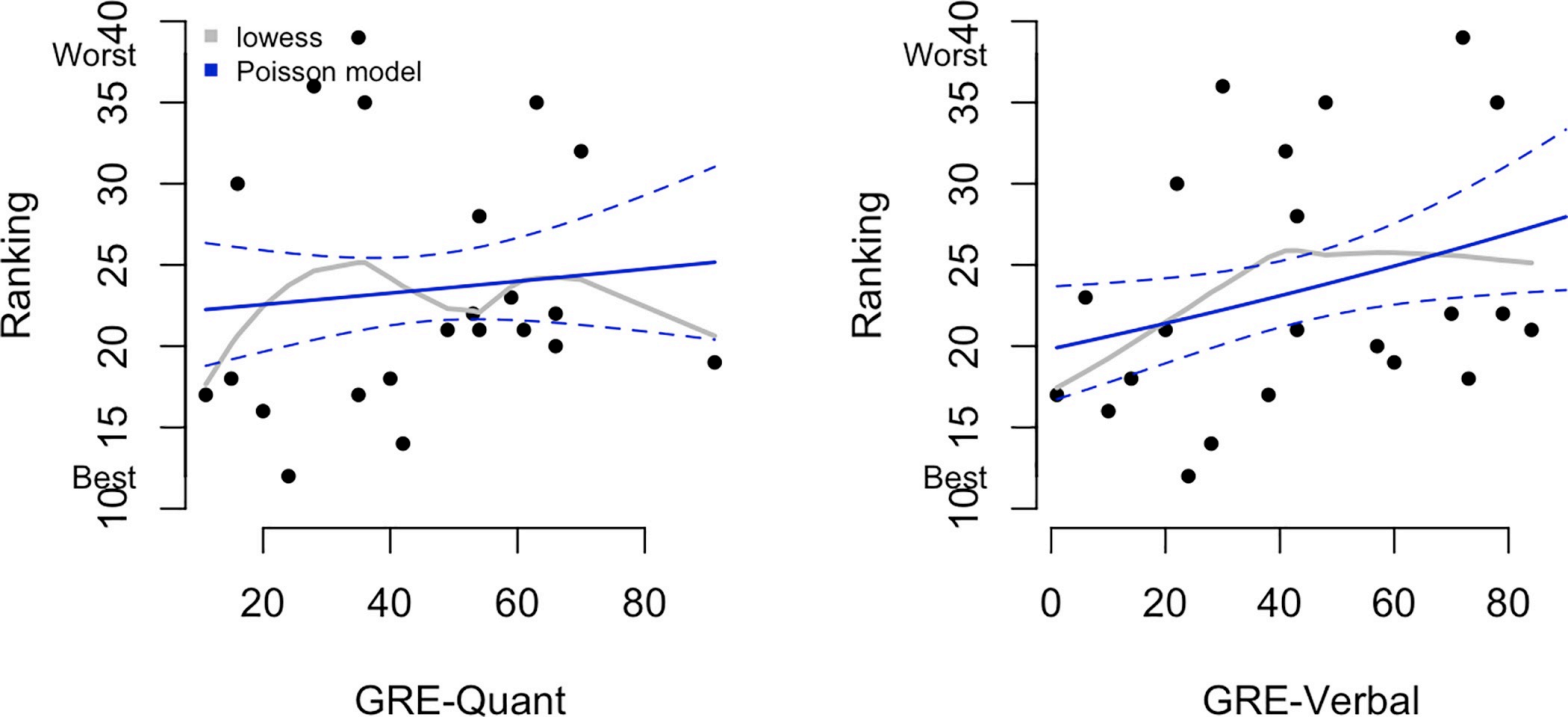
Associations between GRE scores and faculty ranking. Solid curves show the fitted values from the Poisson regression models (dashed lines are 95% confidence intervals) and the grey curves shows lowess smoothers (locally weighted scatterplot smoother).

At the completion of their doctoral training, each faculty mentor is asked to evaluate their PhD student on each of ten questions provided in Table 1. The student is not aware that they are or have been evaluated, and the evaluation is never shared with the student nor used for any other purpose. It is important to note that a lower ranking indicates a better evaluation, with 10 being the highest score possible and 50 the lowest score. Fig 8 (left panel) shows the association between GRE-Q score and faculty ranking. As in the prior figures, solid curves show the fitted values from the Poisson regression models (dashed lines are 95% confidence intervals) and the grey lines show the lowess curves. Corresponding data for GRE-V score and faculty ranking are presented in Fig 8, right panel. In each case the associations were small and actually negative (that is, higher GRE scores were associated with lower faculty rankings). Increasing GRE-Q by 20 percentage points increases (worsens) the expected ranking by 3% (rate ratio = 1.031 with 95% CI 0.914 to 1.164). For instance, for students with GRE-Q scores of 40% and 60%, the expected rankings are 23.267 and 23.994. Likewise, increasing GRE-V by 20 percentage points increases (worsens) the expected ranking by 10% (rate ratio = 1.079 with 95% CI 0.897 to 1.18). For students with GRE-V scores of 40% and 60%, the expected rankings are 23.1 and 24.932.

The data indicate that GRE scores across the entire range of values in this cohort are not predictive of the outcome measures we assessed. We took one final approach–testing for differences in performance measures between the lower and upper quartiles of the GRE scores. To be clear, we compared students with very low scores (<25% GRE-Q or V) to students with very high scores (>75% GRE-Q or V). Although this approach does not use all the data, it would be expected to yield an upwardly biased estimate of the GRE outcome association. The results of such an analysis are shown in Table 4 (for GRE-Q) and Table 5 (for GRE-V). For both tables the first two columns show the mean and standard deviation (SD) of performance measures (number of publications, number of first author publications, months to degree, and faculty ranking) among students in the lower 25th percentile and the upper 25th percentile of GRE score. The third and fourth columns show the difference in mean performance measures between the lower and upper quartiles and the 95% confidence intervals. We see that the point estimates are modest at best, and all confidence intervals include zero as expected. Therefore, even when comparing very low scores, (a range that many graduate schools rarely admit students) to high scores, we do not find evidence that a relationship exists even between the two most likely classes of students.

**Table 4:** Mean (SD) of variables in lower and upper quartiles of GRE-Q and 95% CIs for their difference.

	Lower Q	Upper Q	Difference	95% CI
No. of publications	4.7 (2.5)	5.4 (2.1)	-0.69	(-3.65, 2.28)
No. of first author pubs	1.7 (0.8)	1.8 (0.8)	-0.09	(-1.17, 1.00)
Months to degree	75 (7.6)	72.4 (8.9)	2.57	(-8.8, 13.94)
Faculty ranking	21.5 (9.3)	23.2 (6)	-1.75	(-12.91, 9.41)

The first two columns show the mean and standard deviation (SD) of performance measures among students in the lower 25^th^ percentile and the upper 25^th^ percentile. The third and fourth columns show the difference in mean performance measures between the lower and upper quartiles and the 95% confidence intervals.

**Table 5:** Mean (SD) of variables in lower and upper quartiles of GRE-V and 95% CIs for their difference.

	Lower Q	Upper Q	Difference	95% CI
No. of publications	6 (4.7)	5.4 (2)	0.57	(-3.86, 5)
No. of first author pubs	2.7 (2.1)	2.6 (1.1)	0.14	(-1.86, 2.14)
Months to degree	72.2 (11.1)	69.5 (10.5)	2.69	(-9.9, 15.27)
Faculty ranking	20.8 (5.2)	26.2 (8.6)	-5.33	(-14.76, 4.09)

The first two columns show the mean and standard deviation (SD) of performance measures among students in the lower 25^th^ percentile and the upper 25^th^ percentile. The third and fourth columns show the difference in mean performance measures between the lower and upper quartiles and the 95% confidence intervals.

### Outcomes of the cohort to date

For the 28 students in the cohort analyzed here, the final question we can ask is where are they now? As mentioned earlier, most of the cohort moved on to a postdoctoral position upon PhD completion at a range of research-intensive institutions listed in Table 6. The students in this cohort completed their PhDs between spring 2012 and fall 2018, so some have had time to move to a position beyond the first postdoc. So far after completing their first postdoctoral position, two individuals have moved on to Biopharma, one who is developing a start-up company, one moved to an administrative position at NIH, and one is now a tenure-track assistant professor. At of the time of this writing (November 2018), none of this cohort of 28 students have left science.

**Table 6:** Postdoctoral institutions for IMSD students upon PhD completion.

Harvard University
Mt Sinai Icahn School of Medicine
University of Texas Health Science Center
Northwestern University
Yale University
University of Florida
Vanderbilt University Medical Center
John Hopkins University
National Institutes of Health
Baylor College of Medicine
University of Colorado
Michigan State University
University of Pittsburgh
St Jude Children’s Research Hospital
Case Western University
University of Washington
Charles R. Drew University of Medicine and Science
Vanderbilt University

Institutions where IMSD students who matriculated from 2007–2011 completed first postdocs

## Discussion

As a result of the admissions process adopted by the Vanderbilt IMSD program over a decade ago, we now have a cohort of graduate students whose GRE scores spanned the entire range from 1–91 percentile who have completed the PhD. This analysis includes 28 IMSD students who matriculated into our biomedical research programs from 2007–2011 and completed PhDs beginning in 2012 to fall 2018. In this study, we consistently observed only associations between academic outcomes and GRE scores. Even when accounting for the variability in these estimates (i.e., the width of the 95% CI) we see that the data support at most very minor associations, if any. This is clearly represented by looking at the confidence bands for the regression lines. For example, when modeling the number of first author publications as a function of quantitative GRE score, we found the rate ratio (slope) was 1.154 (95% CI 0.954 to 1.397). This implies that the average change in the number of first author publications is nearly zero even for a large shift in the GRE quantitative percentile. However, the data support changes of approximately [-1 to +1] publication. While not exactly zero, these limited data clearly support the hypothesis that there is only a very minor relationship, if any, between publication and GRE scores. In fact, for verbal scores we observed a very small relationship (not statistically significant from zero) indicating that there is essentially no association in these data. Similar findings can be observed for the other outcome metrics presented here, including total and first author papers, fellowships obtained, time to degree, and faculty evaluations at exit. Importantly, we did observe a statistically significant relationship in the opposite direction with GRE and ranking (better ranked individuals tended to have poorer GRE scores). So, while the overall sample size is small, there is enough precision (or power) in these data to rule out strong meaningful associations if they existed.

We have evaluated verbal and quantitative GRE scores separately in this study, but in actuality a student’s application contains both scores. Perhaps a very low score in one domain (Q or V) may be offset by a high score in the other. In fact, most of the students in this cohort had two reasonably comparable Q and V scores. Of the 28 students, only four had a percentile spread between their two scores of greater than 30. In other words, they were generally either poor test takers or strong ones. Furthermore, only eight of the students who completed PhDs had both GRE-Q and GRE-V scores above the 50^th^ percentile, making it questionable whether the other 20 would have gained admittance to a graduate program that adhered to higher expectations for GRE performance. Five of the 28 students had neither GRE-Q or GRE-V scores above the 30^th^ percentile. We think it unlikely that they would be offered admission to most graduate programs at the time or even to many programs today. Yet, among this group of five is the student who garnered the best (lowest score) faculty evaluation. These outcomes underscore the benefit of giving letters of recommendation, personal statements, and interviews far more weight than GRE scores in making admissions decisions. Our GRE-tolerant approach for increasing the number of students from historically underrepresented groups completing PhDs has been highly successful.

Because all of the students whose performance outcomes are presented here were part of our IMSD program, one concern that can be raised is whether the extra support of this program in some way mitigates the impact low GRE scores would otherwise have. While our IMSD program provides academic support and mentoring to ensure student success, we assert that ensuring student success is of primary importance for all of our students. Indeed, we expect that all graduate programs are striving to provide the academic support and mentoring needed for student success, whether UR or non-UR, IMSD or non-IMSD. Viewing the IMSD program as a source of extra help actually misconstrues its purpose. The Vanderbilt IMSD program exists to build and sustain a **community** of historically underrepresented scholars to provide them with the social and emotional support needed to navigate our majority-white environment. These students experience stereotype threat, imposter syndrome, and implicit biases/microaggressions that impair their sense of belonging and result in high levels of stress. Sometimes this initially manifests itself in underperformance, leading to the erroneous view that historically underrepresented students need extra help. In fact, providing a sense of community and building self-efficacy is what is needed, not extra help, to empower historically underrepresented students to perform to their full potential. Due to stereotype threat and imposter syndrome, URM students may be less likely to seek academic help and, if few in number, form study groups than their non-URM peers. Similar to many programs that promote diversity, we provide intentional opportunities for URM students to gather at journal clubs, data clubs, and review sessions to ensure that students access any help they need, especially through peer mentoring. With social events added to this mix, the cohort becomes invested in the success of all members. Clearly, the sense of community and belonging created by the IMSD program applies to all historically underrepresented students, regardless of their GRE scores.

One reason we have restricted our analysis to IMSD students is because across the range of GRE scores, they all experience the same environmental challenges as non-majority students and have access to the same programmatic supports. However, given the IMSD program is NIH-funded, we have selected a subset of faculty to participate as eligible IMSD mentors based on their mentoring competency. It is possible that the success of the IMSD students across the wide range of GRE scores is due to the strong mentoring they received from their faculty dissertation mentors. Perhaps with good mentoring, GRE scores do not matter. However, to argue that GRE scores are needed to select students because we want them to be successful if they receive sub-optimal mentoring, does not seem like an attractive argument for keeping this standardized test. It may though mean that if graduate programs want the most successful students, they need to focus on faculty mentoring competencies as much, if not more so, than selection criteria for admissions.

Some programs are concerned that without the GREs, less prepared students could be admitted. Understandably, programs do not want to set students up to fail. The student deficit model is a common lens, that students who are not doing well have a lack of skill and/or knowledge that needs to be remedied. If our experience with URM students across the range of GRE scores is any guide, then focusing principally on student competences that need to be improved may prove inadequate without careful attention not only to faculty mentoring skills but also to the culture or environment that students find themselves in. If students are dealing continually with microaggressions and questioning every day if they belong, then anxiety will take its toll on performance. Academic help alone will not solve this problem. This applies whether you bring diversity of gender, gender expression, racial/ethnic, first generation, disadvantaged economic status, or disability to your training environment. There is growing recognition that diversity without inclusion is not enough [5], and that the benefits of diversity in fact depend upon inclusion [6]. Like most, if not all institutions, a truly inclusive climate, although of utmost importance, remains an aspirational goal for us. It is important to consider whether the use of GRE scores actually contributes to the implicit bias, imposter syndrome, microaggressions, and stereotype threat that impair student success. It is well established that historically underrepresented groups on average have lower GRE scores [7,8]. Thus, even students with high GRE scores, but whose visual identity matches groups who historically have lower GRE scores, are at risk of being labeled as low performing when it is erroneously assumed that their GRE scores are probably low. This type of bias is very hurtful, no matter how supportive a community of underrepresented scholars you build. Eliminating GRE scores from the admissions process can help remove a source of bias and thus promote a more inclusive training environment.

The relationship between objective test scores and performance has been a subject of debate for many years [7–13]. In addition to the concerns already described, uncertainty surrounding their predictive ability must be weighed against the cost imposed on applicants to take the test, and the advantages available to a subset of applicants who can prepare extensively ahead of time and/or take the test multiple times to obtain the desired high scores. However, the outcomes of the cohort presented here indicate that non-quantitative measures (letters of recommendation, personal statements, interviews) are capable of selecting successful PhD candidates, even when those candidates have extremely low GRE scores. Subjective measures have their own drawbacks, and we sought to minimize these by having multiple, experienced readers of graduate student applications. We attempted to mediate individual biases by including multiple diverse viewpoints of each student’s potential in reaching a decision to offer admission. Admittedly, this process is time consuming, but the decision of who to train as the next generation of PhD scientists is also arguably one of the most important we make.

The “GRExit” movement is growing, and for those biomedical programs that remain undecided, the data here may be helpful in arriving at a decision on whether or not to continue to require GRE scores for admission. However these decisions turn out, we assert that our GRE-tolerant approach (no score too low) undoubtedly opened doors of opportunity for PhD training at Vanderbilt that may have otherwise remained closed for historically underrepresented students with very low GRE scores. The increased diversity they bring to the community of PhD biomedical scientists will be a benefit for decades to come.

## Supporting information

S1 Table Model results for Fig 3. (DOCX)

S2 Table Model results for Fig 4. (DOCX)

S3 Table Model results for Fig 5. (DOCX)

S4 Table Model results for Fig 6. (DOCX)

S5 Table Model results for Fig 8. (DOCX)

S6 Table Summar statistics of GRE and outcomes data for IMSD and non-IMSD (traditionally admitted) cohorts admitted over the same time period. (DOCX)

S1 Fig Box plots of data from S6 Table. (DOCX)
