# Supplementary material for: The GRE over the entire range of scores lacks predictive ability for PhD outcomes in the biomedical sciences
Source: PLoS One. 2019 Mar 21;14(3):e0201634. doi: 10.1371/journal.pone.0201634 (PMC6428323; doi:10.1371/journal.pone.0201634)
Supplement: S1 Table — (DOCX) [file pone.0201634.s001.docx]

**S1 Table.** (corresponds to Fig 3. Associations between quantitative and verbal GRE scores and total number of publications)

| Table 1a | Rate Ratio | Robust SE | 95% CI | p-value |
| --- | --- | --- | --- | --- |
| Intercept | 0.001 | 0.238 | (0.001, 0.002) | 0 |
| GRE-Q | 1.007 | 0.005 | (0.992, 1.012) | 0.148 |
| Table 1b |  |  |  |  |
| Intercept | 0.002 | 0.271 | (0.001, 0.003) | 0 |
| GRE-V | 1.001 | 0.005 | (0.992, 1.011) | 0.78 |

Results from Poisson regression models looking at the association between GRE-Quantitative and number of publications (Table 1a) and GRE-Verbal and number of publications (Table 1b). The columns show the estimated rate ratios, model robust standard errors, 95% confidence intervals, and p-values.
