# Supplementary material for: The GRE over the entire range of scores lacks predictive ability for PhD outcomes in the biomedical sciences
Source: PLoS One. 2019 Mar 21;14(3):e0201634. doi: 10.1371/journal.pone.0201634 (PMC6428323; doi:10.1371/journal.pone.0201634)
Supplement: S3 Table — (DOCX) [file pone.0201634.s003.docx]

**S3 Table.** (corresponds to Fig 5. Associations between quantitative and verbal GRE scores and months to degree)

| Table 3a | Rate Ratio | Robust SE | 95% CI | p-value |
| --- | --- | --- | --- | --- |
| Intercept | 72.354 | 0.058 | (64.61, 82.02) | 0 |
| GRE-Q | 0.999 | 0.001 | (0.997, 1.002) | 0.612 |
| Table 3b |  |  |  |  |
| Intercept | 71.65 | 0.059 | (63.84, 80.36) | 0 |
| GRE-V | 1 | 0.001 | (0.997, 1.002) | 0.73 |

Results from Poisson regression models looking at the association between GRE-Quantitative and number of months to degree (Table 3a) and GRE-Verbal and months to degree (Table 3b). The columns show the estimated rate ratios, model robust standard errors, 95% confidence intervals, and p-values.
