# Supplementary material for: The GRE over the entire range of scores lacks predictive ability for PhD outcomes in the biomedical sciences
Source: PLoS One. 2019 Mar 21;14(3):e0201634. doi: 10.1371/journal.pone.0201634 (PMC6428323; doi:10.1371/journal.pone.0201634)
Supplement: S4 Table — (DOCX) [file pone.0201634.s004.docx]

**S4 Table.** (corresponds to Fig 6. Boxplots of GRE scores stratified by whether or not the students received a fellowship)

| Table 4a | Odds Ratio | Robust SE | 95% CI | p-value |
| --- | --- | --- | --- | --- |
| Intercept | 2.956 | 1.092 | (0.348, 25.13) | 0.321 |
| GRE-Q | 0.971 | 0.024 | (0.926, 1.017) | 0.207 |
| Table 4b |  |  |  |  |
| Intercept | 0.852 | 0.787 | (0.182, 3.983) | 0.839 |
| GRE-V | 0.997 | 0.016 | (0.967, 1.029) | 0.854 |

Results from logistic regression models looking at the association between GRE-Quantitative and receipt of fellowship (Table 4a) and GRE-Verbal and receipt of fellowship (Table 4b). The columns show the estimated odds ratios, model robust standard errors, 95% confidence intervals, and p-values.
