# Supplementary material for: The GRE over the entire range of scores lacks predictive ability for PhD outcomes in the biomedical sciences
Source: PLoS One. 2019 Mar 21;14(3):e0201634. doi: 10.1371/journal.pone.0201634 (PMC6428323; doi:10.1371/journal.pone.0201634)
Supplement: S5 Table — (DOCX) [file pone.0201634.s005.docx]

**S5 Table.** (corresponds to Fig 8. Associations between GRE scores and faculty ranking)

| Table 5a | Rate Ratio | Robust SE | 95% CI | p-value |
| --- | --- | --- | --- | --- |
| Intercept | 21.878 | 0.177 | (15.45, 30.96) | 0 |
| GRE-Q | 1.002 | 0.003 | (0.996, 1.008) | 0.618 |
| Table 5b |  |  |  |  |
| Intercept | 19.83 | 0.117 | (15.76, 24.95) | 0 |
| GRE-V | 1.004 | 0.002 | (0.999, 1.008) | 0.094 |

Results from Poisson regression models looking at the association between GRE-Quantitative and faculty ranking (Table 5a) and GRE-Verbal and faculty ranking (Table 5b). The columns show the estimated rate ratios, model robust standard errors, 95% confidence intervals, and p-values.
