# Supplementary material for: The GRE over the entire range of scores lacks predictive ability for PhD outcomes in the biomedical sciences
Source: PLoS One. 2019 Mar 21;14(3):e0201634. doi: 10.1371/journal.pone.0201634 (PMC6428323; doi:10.1371/journal.pone.0201634)
Supplement: S6 Table — (DOCX) [file pone.0201634.s006.docx]

**S6 Table. Summary statistics of GRE and outcomes data for IMSD and non-IMSD (traditionally admitted) cohorts.**

|  | IMSD Students | | Non-IMSD Students | |
| --- | --- | --- | --- | --- |
| Variable | **N** | **Summary** | **N** | **Summary** |
| GRE Quantitative | 28 | 48.0 [33.2, 63.0] | 209 | 75 [63, 86] |
| GRE Verbal | 28 | 43.0 [23.5, 62.5] | 209 | 78 [63, 90] |
| No. of publications | 28 | 5.50 [3.75, 7.00] | 208 | 5 [3,7] |
| No. of first author pubs | 28 | 2 [1, 3] | 208 | 2 [1,3] |
| Any fellowship | 28 | 43% (12) | n/a |  |
| F31 fellowship | 28 | 29% (8) | n/a |  |
| Other fellowship: 0 | 28 | 86% (24) | n/a |  |
| 1 |  | 11% (3) | n/a |  |
| 2 |  | 4% (1) | n/a |  |
| Faculty ranking | 22 | 21.0 [18.0, 29.5] | 153 | 19 [13, 28] |
| Months to degree | 28 | 68.6 [64.8, 81.4] | 209 | 67.9 [61.2, 75.6] |

Summary statistics from IMSD students and non-IMSD students. Non-IMSD students were admitted over the same time period by the IGP admissions committee using the traditional process including GRE scores. For continuous variables, *a [b c]* represent the median a, the lower quartile b, and the upper quartile c. *N* is the number of non-missing values. Numbers after percents are frequencies. Fellowship data is only available for IMSD students. 5.7% of the non-IMSD students are historically underrepresented and 100% of the IMSD students are historically underrepresented. Due to the large number of non-IMSD students, publication data are only tracked for 3 years post-graduation because the number of publications by the student/PhD mentor pair beyond this time is very infrequent.
