# Supplementary material for: The GRE over the entire range of scores lacks predictive ability for PhD outcomes in the biomedical sciences
Source: PLoS One. 2019 Mar 21;14(3):e0201634. doi: 10.1371/journal.pone.0201634 (PMC6428323; doi:10.1371/journal.pone.0201634)

**S1 Fig. Box plots of GRE and outcomes data for comparing IMSD and non-IMSD (traditionally admitted) cohorts.** Data from S6 Table are presented graphically.


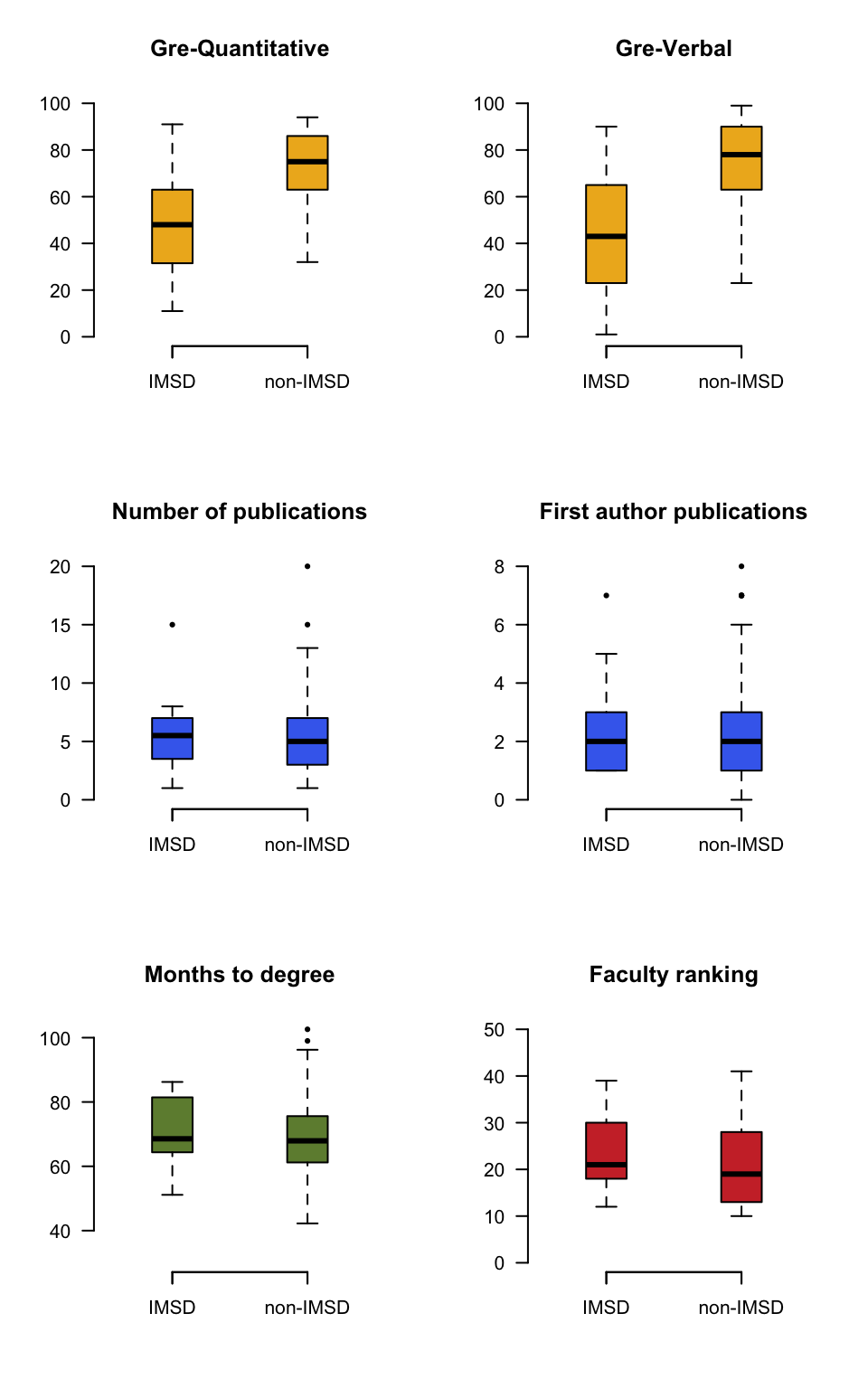

Supplement: S1 Fig — (DOCX) [file pone.0201634.s007.docx]
